# The association between perceived maternal and paternal psychopathology and depression and anxiety symptoms in adolescent girls

**DOI:** 10.3389/fpsyg.2015.00963

**Published:** 2015-07-21

**Authors:** Sanne P. A. Rasing, Daan H. M. Creemers, Jan M. A. M. Janssens, Ron H. J. Scholte

**Affiliations:** ^1^Behavioural Science Institute, Radboud UniversityNijmegen, Netherlands; ^2^GGZ Oost BrabantBoekel, Netherlands; ^3^Praktikon, NijmegenNetherlands

**Keywords:** depression, anxiety, adolescents, girls, parental psychopathology, intergenerational transmission

## Abstract

Exposure to parental depression and anxiety is known to heighten the risk of internalizing symptoms and disorders in children and adolescents. Ample research has focused on the influence of maternal depression and anxiety, but the contribution of psychopathology in fathers remains unclear. We studied the relationships of perceived maternal and paternal psychopathology with adolescents’ depression and anxiety symptoms in a general population sample of 862 adolescent girls (age *M* = 12.39, SD = 0.79). Assessments included adolescents’ self-reports of their own depression and anxiety as well as their reports of maternal and paternal psychopathology. We found that perceived maternal and paternal psychopathology were both related to depression and anxiety symptoms in adolescent girls. A combination of higher maternal and paternal psychopathology was related to even higher levels of depression and anxiety in adolescent girls. Our findings showed that adolescents’ perceptions of their parents’ psychopathology are significantly related to their own emotional problems.

## Introduction

The association between parents’ psychopathology and their children’s psychopathology has been well studied (Hosman et al., 2009; Maybery et al., 2009). Especially the link between parental depression or anxiety and children’s internalizing problems has been a focus of much research (Connell and Goodman, 2002; Goodman and Tully, 2006; England and Sim, 2009; Goodman et al., 2011). It is known that exposure to parental depression or anxiety disorders heightens children’s vulnerability to internalizing symptoms and disorders (Bijl et al., 2002; Lieb et al., 2002) and that particularly mothers’ depression and anxiety are a risk factor for adolescents’ depression or anxiety (Singh et al., 2011). The contribution of fathers’ psychopathology has received increasing attention over the last years and seems to be of equal importance when compared to mothers’ psychopathology (Connell and Goodman, 2002; Ramchandani et al., 2011). To our knowledge, the potential risk to adolescents when both parents suffer from mental health problems compared to one of the parents has not been extensively studied. Therefore, this study focuses on the relationship of parental psychopathology with symptoms of depression and anxiety in adolescents.

Depression and anxiety are common in adolescence, with a prevalence of 5.6% for depression (Costello et al., 2006) and 3–20% for anxiety (Albano et al., 2003). Mental disorders, with depression and anxiety the most prevalent and often not recognized, contribute largely to internalizing problems in young people (Mathews et al., 2011). The disorders and particularly depression, besides their consequences during adolescence and young adulthood, are marked by a recurrent course, and it has been found that the onset of adult depression and anxiety often emerges during adolescence (Birmaher et al., 1996; Albano et al., 2003; Costello et al., 2006; Beesdo et al., 2009; Dobson et al., 2010). Knowing that internalizing disorders have the highest lifetime prevalence, estimated to be 3.3–21.4% for mood disorders and 4.8–31.0% for anxiety disorders (Kessler et al., 2007), it is important to identify risk factors for development of these disorders in adolescents. It is known that adolescent girls are more vulnerable to develop a depressive or anxiety disorder than boys (Nolen-Hoeksema, 2001; Garber et al., 2002). Also elevated symptoms of depression or anxiety symptoms are more present in girls than in boys during adolescence (Hankin and Abramson, 2001). Because girls have a higher risk on developing depression or anxiety, we focused specifically on adolescent girls.

Children of parents with a mental disorder have an elevated risk of developing psychopathology themselves (Merikangas et al., 1998; Lieb et al., 2002; Micco et al., 2009). More specifically, in case of parental depression, children are three times more likely to develop depression compared to children of healthy parents (Weissman et al., 2006). In case of parental anxiety, children are two to seven times more likely to develop an anxiety disorder (Beidel and Turner, 1997). Thus, intergenerational transmission of mental health problems is rather consistent (Goodman and Gotlib, 1999). Further, research showed that there was no interaction between gender and parental depression and that transmission of depression from parents to children was comparable for boys and girls (Bouma et al., 2008). Models describing the intergenerational transmission refer to several factors, such as heritability, exposure to parental maladaptive behavior and cognitions, and exposure to a stressful dysfunctional family situation (Goodman and Gotlib, 1999; Garber and Cole, 2010). This, however, describes the contribution of parents in general and does not explain the extent to which mothers or fathers, uniquely and in combination, influence the development of depression or anxiety in adolescents.

The maternal and paternal influences on the normal development of children have been studied extensively, and it is known that both parents have their own unique contribution (Lamb, 2010). In the development of children’s psychopathology, fathers’ contribution has long been underestimated and therefore not well understood. Most adolescents’ problems were attributed to the influence of mothers (Phares, 1992). Recently, the contribution of fathers has been increasingly recognized, although mostly in the development of externalizing problems rather than internalizing problems (Phares and Compas, 1992).

Beside the influence of mothers’ and fathers’ psychopathology as single risk factor, the presence of paternal psychopathology has also been conceptualized as a moderator of maternal psychopathology as suggested based on previous studies (Weissman et al., 1984; Goodman and Gotlib, 1999). This implies that psychopathology of fathers may additionally increase the risk for psychopathology in children, especially when mothers suffer from mental health problems. However, whether having two parents – instead of one – with psychopathology indeed increases the level of symptoms in adolescents needs to be studied more carefully.

As a measure of parental psychopathology, earlier studies on the relationship between parental psychopathology and children’s psychopathology used mostly the symptom level or clinical diagnosis as reported by the parents. However, it has been suggested that children are best informants of their own internalizing symptoms (Bird et al., 1992; Kazdin, 1994), and several studies used children as informants of parental characteristics as well, such as parent–child relationships and parenting behavior (Forehand and Nousiainen, 1993). To our knowledge, no studies have used adolescents’ perceptions to further unravel the relationships between maternal, paternal, and children’s mental health. Thus, this study would contribute to the understanding of whether parental psychopathology can be measured based on adolescents’ perception, and whether the same relationships hold as those between parent-rated parental psychopathology and adolescents’ psychopathology.

The first aim of the current study was to examine whether parental psychopathology, as perceived by adolescent girls, was related to adolescent psychopathology. More specifically, we studied whether maternal and paternal psychopathology were related to depression and anxiety symptoms in adolescent girls. The second aim was to explore whether the presence of both maternal and paternal psychopathology, rather than only maternal or paternal symptomatology, was related to higher depression and anxiety symptoms in adolescent girls. We hypothesized that higher perceived maternal and paternal psychopathology were separately related to a higher level of depressive and anxiety symptoms in adolescent girls, and that maternal and paternal psychopathology would have an additive effect on depression and anxiety symptoms in adolescent girls.

## Materials and Methods

For this study, data of the screening procedure of a Dutch randomized controlled trial (Dutch Trial Register NTR3720) on the prevention of depression and anxiety in adolescent girls with high familial risk were used (Rasing et al., 2013). The medical ethics committee CMO Region Arnhem-Nijmegen, The Netherlands, has approved this study.

### Participants and Procedure

Female students in the first and second grade of secondary school received written information about the study together with an opt-out form, which allowed them and their parents to refuse the participation. After passive consent was received, 862 female adolescents completed questionnaires about symptoms of depression or anxiety and about their perceptions of psychopathology in their parents. These students were selected from five schools ranging from vocational education up to pre-university education in rural area as a representative sample of the general adolescent female population. The age of the adolescents ranged from 11 to 15, with a mean age of 12.39 (SD = 0.79). Most adolescents were of Dutch origin (96.6%).

### Measures

#### Depression

The Dutch version of the Children’s Depression Inventory 2 (CDI 2; Kovacs, 2012), which consists of 28 items, was used to measure depression symptoms. Each item consists of three statements rated in severity from 0 to 2. Sample statements include, “Sometimes I feel sad,” “Most of the times I feel sad,” and “I always feel sad.” Cronbach’s alpha was 0.87.

#### Anxiety

The Dutch version of the Spence Children’s Anxiety Scale (SCAS; Spence, 1997) was used to measure anxiety symptoms. This 44-item self-report questionnaire measures the frequency of symptoms on a 4-point scale ranging from never to always. Sample statement is, “I worry about things.” Cronbach’s alpha was 0.85.

#### Adolescents’ Perception of Parental Psychopathology

Students responded to seven statements about parental psychopathology for both mother and father. Adolescents indicated whether the following statements are true for their parents: “My parent received treatment from a psychologist or psychiatrist,” “My parent had a depressed mood for more than 2 weeks,” “My parent had a decreased interest or pleasure in most or all activities,” “My parent had a period of fatig or loss of energy,” “My parent had excessive worry and anxiety about general events for at least 6 months,” “My parent had excessive and unreasonable fear of a specific situation (e.g., elevators) or object (e.g., spiders)” and “My parent had recurrent panic attacks, with or without fear to leave his/her home or safe environment.” In all statements, wording of parent was replaced by either mother or father. Answers were rated as not present (0) or present (1). Because the seven items were related to several concepts – three items were related to parental depression, three to parental anxiety, and one to parental treatment – we could not assume that the seven items were highly related to each other and that the seven items could be interpreted as a unidimensional scale. Therefore, we did not compute a classic Cronbach’s alpha. To assess a general indication for parental psychopathology, we counted the number of items for which their adolescent daughters indicated parental psychopathology.

### Statistical Analyses

First, means, SDs, and bivariate correlations were computed for all study variables. Second, hierarchical regression analyses were performed to examine the relationships and the interaction of maternal and paternal psychopathology with adolescents’ depression and anxiety. All predictor variables were continuous and centered in all analyses before testing interactions (Aiken and West, 1991).

In the first regression analysis, we used adolescent depression as an outcome variable, and entered perceived maternal psychopathology as predictor variable in step 1, perceived paternal psychopathology as predictor variable in step 2 and their interaction in step 3. In the second analysis, adolescent anxiety was the outcome variable and again, we entered the perceived maternal psychopathology as predictor variable in step 1, perceived paternal psychopathology as predictor variable in step 2 and their interaction in step 3.

## Results

### Descriptive Statistics and Correlations

Descriptive statistics were computed for adolescents’ depression symptoms (*M* = 8.13, SD = 6.06), anxiety symptoms (*M* = 26.69, SD = 13.89), perceptions of maternal psychopathology (*M* = 0.89, SD = 1.18) and perceptions of paternal psychopathology (*M* = 0.52, SD = 1.00). Bivariate correlations among study variables (**Table 1**) showed that adolescents’ depression and anxiety symptoms were highly correlated. Further, adolescents’ depression and anxiety symptoms were positively related to the adolescents’ perceptions of maternal and paternal psychopathology. Additionally, perceived maternal and paternal psychopathologies were positively related.

**Table 1:** Correlations between model variables.

	(2)	(3)	(4)
(1) Depression symptoms adolescents	0.72***	0.34***	0.25***
(2) Anxiety symptoms adolescents		0.34***	0.26***
(3) Perceived maternal psychopathology			0.43***
(4) Perceived paternal psychopathology			

***p < 0.001.

**FIGURE 1:**
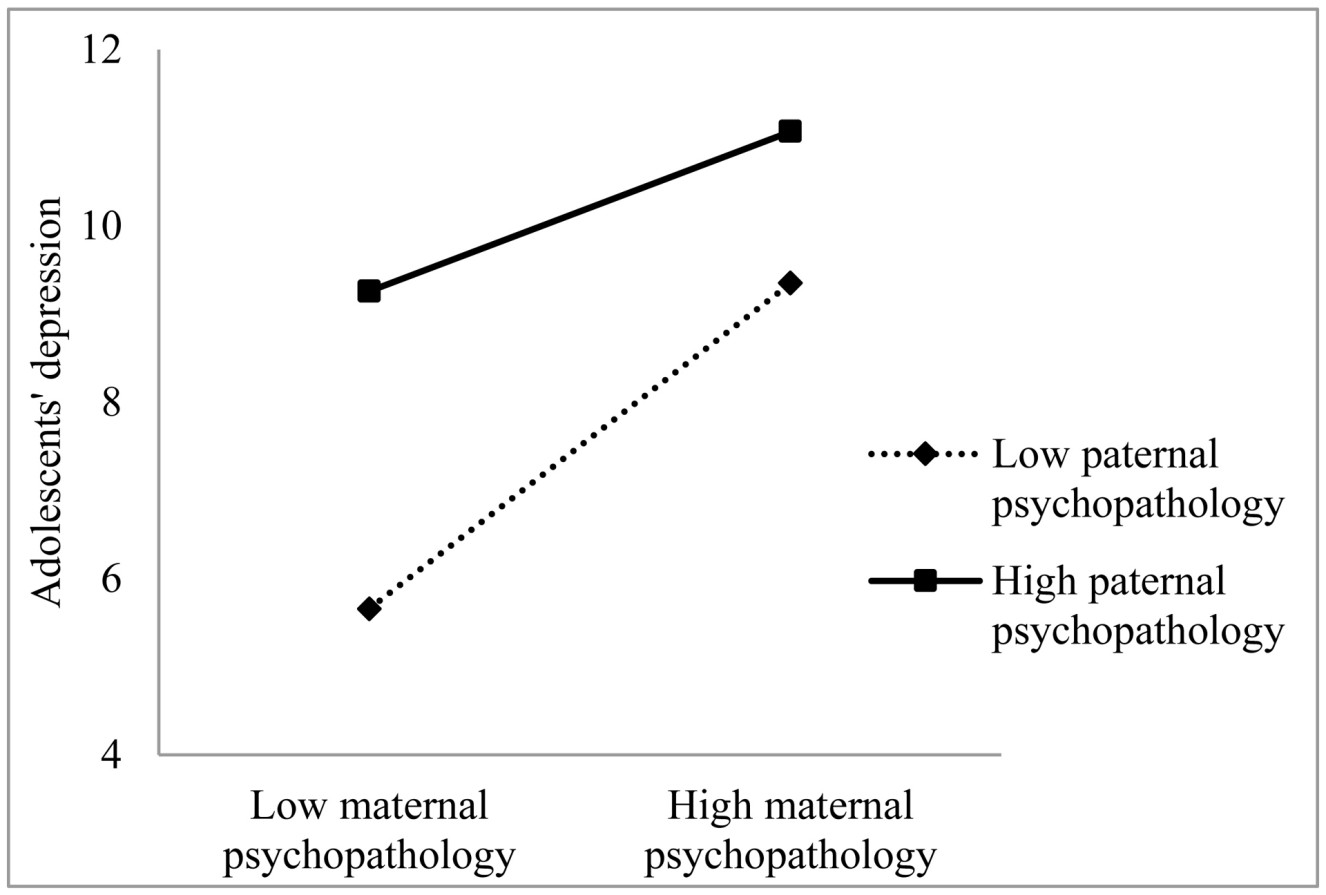
**Depression symptoms in adolescent girls predicted by the interaction of perceived maternal and paternal psychopathology**.

As can be seen in **Table 2**, perceived maternal and paternal psychopathology were significant predictors of depression symptoms in adolescents. Additionally, interaction between perceived maternal and paternal psychopathology was a significant predictor of depression symptoms in adolescents. This interaction can also be seen in **Figure 1**, where the predictors maternal and paternal psychopathology are divided in low (below mean) and high (above mean).

**Table 2:** Regression analyses of perceived maternal and paternal psychopathology and their interaction effect on depression symptoms in adolescent girls.

	*B*	SE	β	*t*	*F*(F-change)	Δ*R*^2^
*Step 1*					104.78	0.11***
Perceived maternal psychopathology	1.71	0.17	0.33	10.24***		
*Step 2*					59.76 (13.21)	0.01***
Perceived maternal psychopathology	1.42	0.18	0.28	7.71***		
Perceived paternal psychopathology	0.79	0.22	0.13	3.63***		
*Step 3*					44.62 (12.70)	0.01***
Perceived maternal psychopathology	1.59	0.19	0.31	8.42***		
Perceived paternal psychopathology	1.13	0.24	0.19	4.78***		
Interaction perceived maternal psychopathology * perceived paternal psychopathology	-0.41	0.12	-0.14	-3.56***		

***p < 0.001.

**FIGURE 2:**
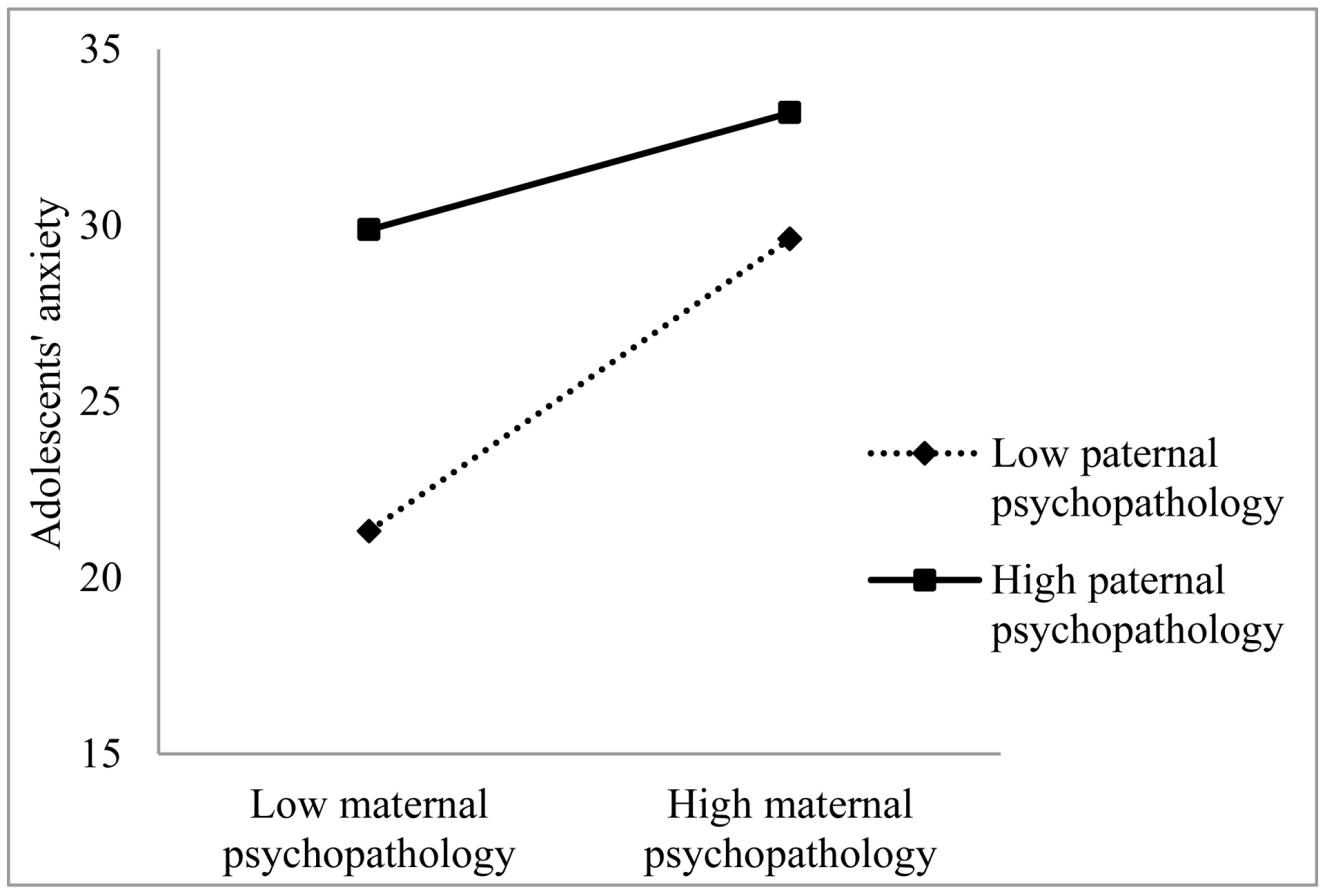
**Anxiety symptoms in adolescent girls predicted by the interaction of perceived maternal and paternal psychopathology**.

The same relationships were found between perceived maternal and paternal psychopathology and anxiety symptoms in adolescents, as can be seen in **Table 3**. The interaction between perceived maternal and paternal psychopathology also significantly predicted adolescents’ anxiety symptoms. This interaction can also shown in **Figure 2**, where again the predictors maternal and paternal psychopathology are divided in low (below mean) and high (above mean).

**Table 3:** Regression analyses of perceived maternal and paternal psychopathology and their interaction effect on anxiety symptoms in adolescent girls.

	*B*	SE	β	*t*	*F*(F-change)	Δ*R*^2^
*Step 1*					113.32	0.12***
Perceived maternal psychopathology	4.08	0.38	0.34	10.65***		
*Step 2*					64.28 (13.54)	0.01***
Perceived maternal psychopathology	3.40	0.42	0.29	8.07***		
Perceived paternal psychopathology	1.84	0.50	0.13	3.69***		
*Step 3*					45.03 (5.81)	0.01*
Perceived maternal psychopathology	3.67	0.44	0.31	8.44***		
Perceived paternal psychopathology	2.36	0.54	0.17	4.35***		
Interaction perceived maternal psychopathology * perceived paternal psychopathology	-0.64	0.27	-0.09	-0.09*		

*p < 0.05, ***p < 0.001.

This means that higher perceived maternal psychopathology was associated with higher levels of adolescents’ depression and anxiety symptoms and that higher perceived paternal psychopathology was also associated with more depression and anxiety symptoms in adolescents. Moreover, the combination of higher perceived maternal and paternal psychopathology had an additive effect on symptoms of depression and anxiety in adolescents.

## Discussion

The present study examined whether perceived parental psychopathology was related to symptoms of depression and anxiety in adolescent girls. Furthermore, we explored whether the presence of both perceived maternal and perceived paternal psychopathology was related to higher depression and anxiety symptoms in adolescent girls compared to when only one of the parents was reported to show psychopathology.

Our findings showed that perceived maternal and paternal psychopathology were related to depression and anxiety symptoms in adolescent girls, in accordance with previous studies, which demonstrated the same relationships (McClure et al., 2001; Brennan et al., 2002). We also found that a combination of higher perceived maternal psychopathology and higher perceived paternal psychopathology was related to even higher depression and anxiety symptoms in adolescent girls. This is consistent with previous studies, which showed relations among maternal, paternal, and offspring symptoms (Klein et al., 2005; Renk et al., 2007). In Goodman and Gotlib’s (1999) model of intergenerational transmission, paternal mental health is described as a moderator in the development of the child’s psychopathological symptoms. Healthy fathers could compensate for genetic risk and provide healthy cognitions, behavior, and affect. They could provide substitute caregiving and could provide support for mothers. When only one parent suffers from mental health problems, the healthy parent can be seen as a protective factor by acting as a positive role model (Goodman and Gotlib, 1999; Connell and Goodman, 2002; Ramchandani and Psychogiou, 2009). When both parents show symptoms of psychopathology; thus, the protective factor is not present, children are likely to experience more psychopathology. Earlier studies also confirmed that parental psychopathology in both parents had an additive effect and resulted in a higher symptom level symptoms in children (Goodman et al., 1993; Dierker et al., 1999).

In this study, we used adolescent girls’ perceptions to assess parental psychopathology. Our findings showed strong similarities with earlier studies, which used parents as informants of parental psychopathology. Given our findings, using adolescent girls’ perceptions seems a promising way to measure parental psychopathology. However, there also might be some limitations using adolescents as informants; according to the distortion hypothesis (Richters and Pellegrini, 1989), the informants’ psychopathology influences the report of symptoms of a different person. Richters and Pellegrini (1989) and Richters (1992) studied the accuracy of depressed mothers’ reports informants of their child’s symptoms of psychopathology. They hypothesized that a negative perceptual bias related to mothers’ own depression results in over reporting of their children’s symptoms. This might also be the case for our adolescents’ reports of their parents’ psychopathology. Another limitation is that we studied these relationships only in adolescent girls. The relationship between perceived maternal psychopathology and perceived paternal psychopathology with depression and anxiety symptoms might be different in adolescent boys than in girls, because girls are known to be more responsive to depression than boys (Nolen-Hoeksema, 2001; Garber et al., 2002). Although earlier research suggested that transmission of depression from parents to children was comparable for boys and girls, future studies should also focus on boys instead of only girls.

Since the present study used a cross-sectional design, we were not able to test causal pathways in the intergenerational transmission of psychopathology. Future studies should therefore examine these processes utilizing a longitudinal study design in order to determine how these processes develop and in what sequence. Further, studies should consider using disorder-specific questionnaires. For our measures of perceived parental psychopathology, we used seven statements related to three different concepts, parental depression, anxiety and treatment instead of using unidimensional scales for each of these three concepts. Measuring parental depression and anxiety using for each a unidimensional instrument may result in more valid and reliable measurements of specific types of parental psychopathology.

The clinical implication of this screening method is that adolescents at risk for depression or anxiety can be identified in clinical practice. This makes it possible to intervene early and to prevent them from developing a clinical disorder. Selecting an appropriate depression and anxiety prevention program and testing its effectiveness should be considered as the next step.

## Conclusion

The present study showed that perceived maternal and paternal psychopathology were related to symptoms of depression and anxiety in their adolescent daughters. Depression and anxiety symptoms in adolescent girls were even higher when both parents had higher psychopathology, which underlines the relevance of parental psychopathology in both parents. The causal pathways, however, are unclear and need to be studied in longitudinal studies. Future research should examine how the intergenerational transmission of depression and anxiety unfolds over time as well as from an earlier age through adolescence into young adulthood.

## Conflict of Interest Statement

The authors declare that the research was conducted in the absence of any commercial or financial relationships that could be construed as a potential conflict of interest.
